# Gastric cancer and pleomorphic liposarcoma: A Case Report of synchronous dual primary malignancies

**DOI:** 10.3389/fonc.2026.1837427

**Published:** 2026-05-25

**Authors:** Tingting Hui, Lixia Lin, Zhong Yang

**Affiliations:** 1 Department of LanZhou Cadre Retirement, Gansu Province Military Region, Lanzhou, China; 2 Department of Surgical Oncology, Gansu Provincial Hospital, Lanzhou, China; 3 The First Clinical Medical College of Lanzhou University, Lanzhou University, Lanzhou, China

**Keywords:** case report, gastric cancer, multiple primary malignant neoplasms (MPMN), pleomorphic liposarcoma (PLPS), prognosis

## Abstract

A 62-year-old male presented with a 9-month history of intermittent epigastric distension and pain, accompanied by acid reflux, nausea, vomiting, melena, and weight loss. Physical examination revealed epigastric tenderness. Laboratory tests showed hemoglobin of 94.0 g/L and serum albumin of 22.55 g/L. Gastroscopy identified gastric cancer in the antrum with gastric retention. Pathological biopsy of the antrum confirmed poorly differentiated adenocarcinoma. Colonoscopy revealed multiple small colonic polyps (maximum diameter 5 mm). Contrast-enhanced computed tomography (CT) of the upper abdomen demonstrated an approximately 6 cm lesion in the gastric antrum and a mesenchymal malignant tumor measuring approximately 10 cm in diameter in the left hepatorenal space, along with ascites in the abdominal and pelvic cavities. The patient received preoperative nutritional support, which improved his anemia, hypoalbuminemia, and overall physical condition. Under general anesthesia, he underwent distal gastrectomy with D2 lymph node dissection and Billroth II reconstruction, combined with resection of the retroperitoneal tumor. Postoperative pathology confirmed the gastric lesion as an ulcerative poorly differentiated adenocarcinoma and the retroperitoneal tumor as a pleomorphic liposarcoma (PLPS). The patient recovered well and was discharged. For the gastric cancer, he received 6 cycles of adjuvant chemotherapy with the SOX regimen (Oxaliplatin plus S-1). Follow-up examinations, including gastroscopy and contrast-enhanced CT of the upper abdomen, were performed at 3, 6, 12, 18, 24, 30, 36, 42, and 48 months postoperatively. No evidence of tumor recurrence was detected during this follow-up period. This paper reports a concurrent case of gastric cancer and PLPS, detailing the case characteristics, diagnostic and therapeutic procedures, and postoperative follow-up. The purpose of this study is to analyze the features of this Multiple primary malignant neoplasms (MPMN) case and provide a reference for selecting appropriate clinical treatments to improve patient prognosis.

## 1 Introduction

Multiple primary malignant neoplasms (MPMN) refer to the occurrence of two or more histologically distinct primary tumors in a single patient, either simultaneously or sequentially. These tumors may arise within different sites of the same organ or system, or within different organs or systems altogether (1). Tumors diagnosed within an interval of <6 months are generally classified as synchronous MPMN, while those occurring >6 months apart are termed metachronous MPMN (2). Although reports of gastric cancer occurring within MPMN are increasing, its synchronous presentation with pleomorphic liposarcoma (PLPS) is exceptionally rare. This article details the clinical characteristics, diagnostic and therapeutic management, and postoperative follow-up of a simultaneous gastric cancer and PLPS case, aiming to provide a reference for future clinical practice.

## 2 Case description

A 62-year-old male presented with a 9-month history of intermittent epigastric pain and abdominal distension. Associated symptoms included acid regurgitation, nausea, vomiting, and melena. The patient reported significant anorexia with an unintentional weight loss of 15 kg. There was no personal history of malignancy or relevant family history. Physical examination revealed a flat abdomen with mild epigastric tenderness. No rebound tenderness, guarding, hepatosplenomegaly, or palpable masses were detected. Murphy’s sign was negative. Routine blood tests revealed leukopenia (WBC 2.6 × 10^9^/L), anemia (Hb 94.0 g/L), and a normal platelet count (291 × 10^9^/L). Serum biochemistry revealed hypoproteinemia(total protein 58.94 g/L) with severe hypoalbuminemia(albumin 22.55 g/L). Serum tumor markers were within normal limits. Upper endoscopy demonstrated reflux esophagitis, features suspicious for gastric carcinoma, and gastric food retention. Histopathological examination of biopsy specimens confirmed poorly differentiated adenocarcinoma (**Figure 1**). Colonoscopy revealed multiple colorectal polyps, the largest measuring 0.5 cm in diameter.

**Figure 1:**
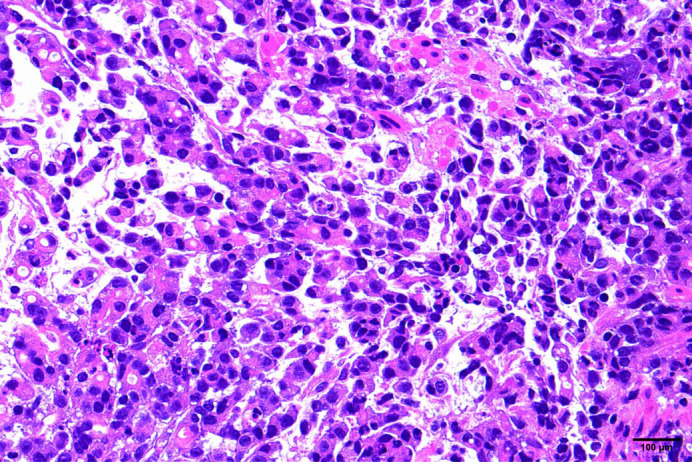
Histopathological examination of the gastric antral biopsy revealed poorly differentiated adenocarcinoma.

Computed tomography (CT) revealed a 6-cm gastric antral lesion radiologically compatible with gastric carcinoma (clinical stage T2N0Mx). Additionally, a 10-cm mass was identified in the right retroperitoneal space, adjacent to the right hepatic lobe and right kidney (**Figure 2**).

**Figure 2:**
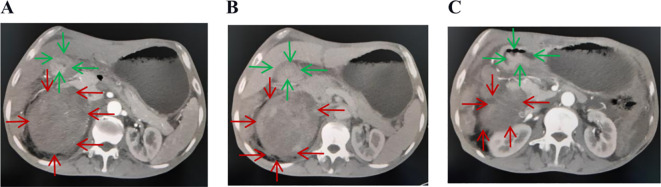
(**A–C**) Gastric and retroperitoneal tumors on sequential CT slices, with red and green arrows marking the retroperitoneal and gastric antral lesions, respectively.

After nutritional optimization and albumin replacement, the patient underwent combined resection following multidisciplinary consensus: distal gastrectomy (D2 lymphadenectomy) with Billroth II reconstruction plus retroperitoneal mass excision. Recovery was uneventful. Serial follow-up demonstrated no recurrence at 6 months, with sustained disease-free status at 48-month final evaluation. The resection specimen is shown in **Figure 3**.

**Figure 3:**
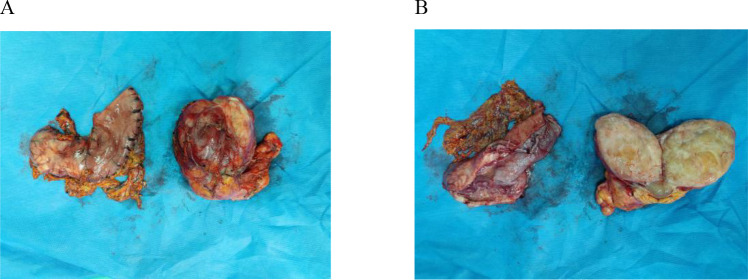
Gross specimens include distal gastric **(A)** and retroperitoneal tumors **(B)**.

Histological examination of the distal stomach revealed an ulcerated, poorly differentiated gastric adenocarcinoma. Located at the lesser curvature, the ulcerative tumor measured 9×8×2 cm and invaded the full thickness of the gastric wall. The adenocarcinoma demonstrated perineural invasion. No lymphovascular invasion (tumor thrombus) was identified, and the greater omentum showed no evidence of tumor involvement. All 36 examined lymph nodes were negative for metastasis (0/36).Immunohistochemical and molecular pathological examinations revealed the following: Negative for CD31 (no vascular invasion), D2-40 (no lymphatic invasion), chromogranin A (CgA), synaptophysin (Syn), CD56, HER2/neu (HER2), Helicobacter pylori (HP), and EBER; positive for S-100 (indicating neural invasion), CDX-2, MLH1, MSH2, MSH6, PMS2, cytokeratin pan (CKP), cytokeratin 8-18 (CK8-18), CD45, and prostate-specific antigen (PSA); p53 wild-type expression pattern; Ki-67 proliferation index 80% (**Figure 4**).The histopathological assessment according to the AJCC staging system classified this gastric carcinoma as pT3N0M0.The hematoxylin-eosin staining picture is shown in **Figure 5A**.

**Figure 4:**
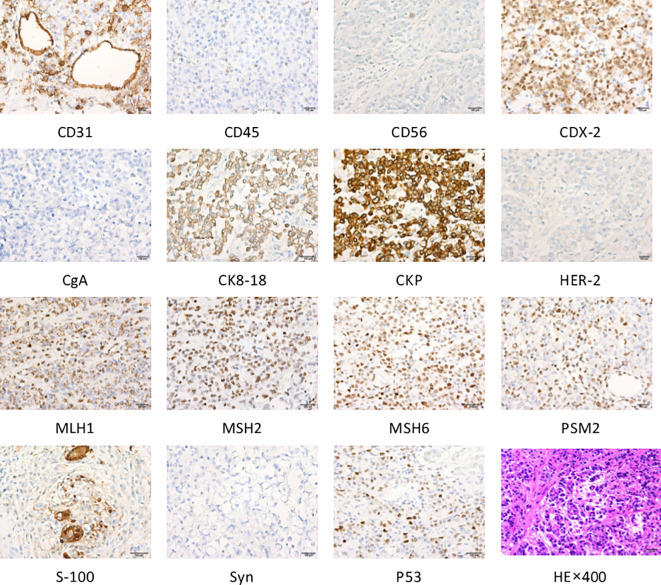
Immunohistochemical molecular map of gastric cancer.

**Figure 5:**
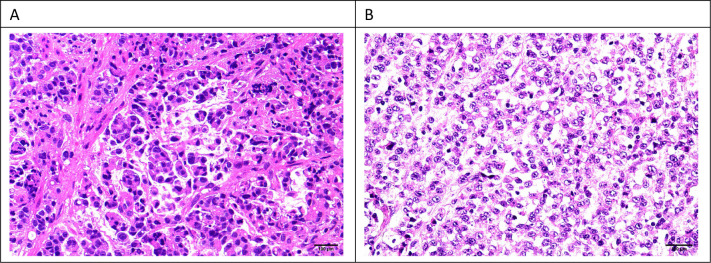
Pathological gastric cancer **(A)** and pathological retroperitoneal pleomorphic liposarcoma **(B)**.

The retroperitoneal tumor was identified as a pleomorphic liposarcoma measuring 15 × 13 × 10 cm. Immunohistochemical and molecular pathological analysis demonstrated the following: negative for cytokeratin pan (CKP), cytokeratin 8-18 (CK8-18), CD56, and carcinoembryonic antigen (CEA); positive for S-100 protein and vimentin; MDM2-negative; CDK4-positive; and a Ki-67 proliferation index of 5% (**Figure 6**). Representative hematoxylin and eosin (HE)-stained tissue sections of the pleomorphic liposarcoma are shown in **Figure 5B**.

**Figure 6:**
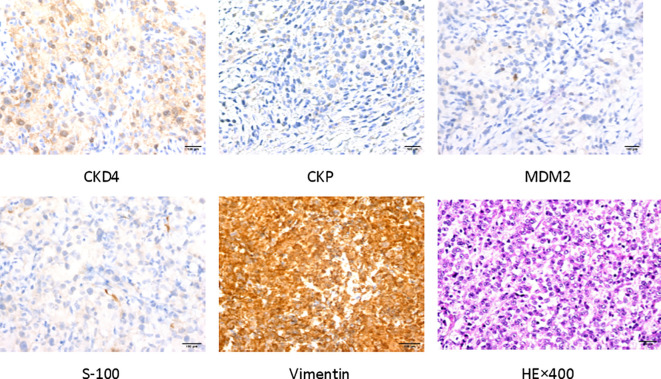
Immunohistochemical molecular map of pleomorphic liposarcoma.

The patient recovered well postoperatively and was discharged. Adjuvant chemotherapy with the SOX regimen (Oxaliplatin plus S-1) was administered. Follow-up examinations were conducted at 3, 6, 12, 18, 24, 30, 36, 42and 48 months postoperatively. These evaluations included patient history review, physical examination, tumor marker assays, gastroscopy, and contrast-enhanced CT of the upper abdomen. Comprehensive assessment based on these findings revealed no evidence of tumor recurrence (**Figure 7**).

**Figure 7:**
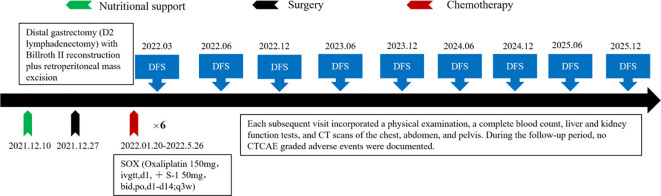
A timeline diagram illustrating the patient’s entire treatment course.

## 3 Discussion

There has been an increase in reported cases of MPMN in recent years. Our department previously documented a case of concurrent gastric and endometrial cancer treated with surgical resection, resulting in favorable patient outcomes (3). In the present case, following multidisciplinary team discussion, radical distal gastrectomy combined with retroperitoneal tumor resection was performed. No evidence of tumor recurrence was observed at the 6-month postoperative follow-up. Critically, the patient remained alive with no signs of recurrence at the last follow-up 48months postoperatively, indicating that surgical resection may confer benefit for patients with synchronous gastric cancer and PLPS.

When diagnosing gastric lesions, it’s crucial to distinguish them from benign gastric ulcers, which typically exhibit a classic ulcer shape with inward-converging surrounding mucosa and lack malignant cell traits, gastric lymphomas that differ pathologically from adenocarcinomas with distinct cell morphologies and immunohistochemical profiles, and gastrointestinal stromal tumors boasting unique immunohistochemical markers like positive CK8–18 and CKP; for retroperitoneal tumors, differentiation is necessary from lipomas with regular, atypia-free cell morphology, spindle-shaped leiomyosarcoma cells with immunohistochemical markers distinct from PLPS, and malignant fibrous histiocytomas showing varied cell morphologies but clearly differing immunohistochemical features from PLPS; through detailed pathological and immunohistochemical analysis, we confirmed the diagnoses of ulcerative poorly differentiated gastric adenocarcinoma and retroperitoneal PLPS. PLPS represents a rare and aggressive liposarcoma subtype, accounting for approximately 10% of all liposarcomas (4, 5). It typically arises within the soft tissues of the retroperitoneum or extremities and is characterized by diagnostic challenges and poor prognosis. Histopathologically, PLPS exhibits pleomorphic features including bizarre mononuclear or multinucleated giant cells alongside lipoblasts demonstrating marked nuclear atypia, hyperchromasia, and vacuolated cytoplasm (6). Immunohistochemical findings are variable: while CD34 (+), S-100 (+), and vimentin (+) (7) have been reported, MDM2 expression shows significant heterogeneity—demonstrating negativity in some cases (8) and strong positivity in others (9). This immunohistochemical inconsistency further complicates definitive diagnosis.

PLPS predominantly affects middle-aged and elderly populations, with a reported age range of 27–95 years and a median age of 54 years (10). No standardized treatment protocol currently exists. Notably, conventional chemotherapy has not demonstrated significant improvement in overall survival rates (11–13). Given the tumor’s high malignant potential, propensity for local recurrence, and metastatic risk, wide-margin surgical resection remains the cornerstone of treatment (14), necessitating rigorous postoperative surveillance.

Most diagnoses of synchronous PLPS are incidental, with presenting symptoms typically attributable to the coexisting primary malignancy rather than the liposarcoma itself. This underscores the critical importance of comprehensive imaging evaluation in middle-aged and elderly cancer patients. Additionally, genetic counseling is indicated for patients with hereditary tumor syndromes, such as those with concurrent breast malignancies (15, 16).

Compared to solitary primary tumors, MPMN portends a significantly poorer prognosis (17). Consequently, clinicians must maintain heightened vigilance for gastric cancer occurring within the context of MPMN. Early detection and accurate diagnosis are paramount for optimizing patient outcomes, necessitating rigorous digestive system screening protocols.

For patients presenting with synchronous gastric cancer and PLPS, management should be individualized based on tumor-specific characteristics including histologic subtype, staging, and patient performance status. Multidisciplinary team consultation—incorporating gastrointestinal surgery, surgical oncology (sarcoma specialists), medical oncology, and radiation oncology—is essential for therapeutic decision-making. Neoadjuvant chemotherapy may be considered for locally advanced gastric cancers to facilitate R0 resection (18), with emerging evidence supporting its potential role in downsizing large PLPS lesions. Ultimately, maximal safe resection combined with adjuvant radiotherapy represents the cornerstone of local control in PLPS management.

## 4 Conclusion

While no standardized treatment paradigm currently exists for PLPS, surgical resection has demonstrated clinical benefit in our experience managing synchronous gastric cancer and PLPS, correlating with improved disease-free survival outcomes. For patients with PLPS, we recommend concurrent gastroscopy and colonoscopy. When clinically indicated, PET-CT may be utilized for systematic evaluation. In cases of MPMN, extensive systemic surveillance should be prioritized during follow-up, extending beyond gastrointestinal assessment.

## Data Availability

The original contributions presented in the study are included in the article/supplementary material. Further inquiries can be directed to the corresponding author.
